# Toxoplasma’s Taste for Exotic Fat

**DOI:** 10.1371/journal.pbio.1002289

**Published:** 2015-11-13

**Authors:** Roland G. Roberts

**Affiliations:** Public Library of Science, Cambridge, United Kingdom

## Abstract

A new study reveals that an exotic lipid, phosphatidylthreonine, makes up a substantial proportion of the membrane of a widespread human parasite and is essential for its virulence. Read the Research Article.

Every cell in nature is bound by a gossamer membrane with relative dimensions akin to those of a rubber balloon filled with water. These membranes are made of two back-to-back layers comprising a blend of jiggling lipid molecules—the so-called “lipid bilayer”—and in most organisms, the predominant ingredients in the lipid blend are the glycerophospholipids.

These molecules each comprise two long fatty acids buried deep in the interior of the bilayer, connected to a water-loving glycerol and phosphate boss. But attached to the phosphate group are a range of different head-group decorations exposed either to the cell’s interior or to the extracellular space; it is these that, via their various shapes, sizes, and charges, determine the characteristics of individual lipids and, in turn, of the biological membranes. Only a limited repertoire of such head-groups has been identified so far in nature—the common ones are the alcohol amines ethanolamine and choline, the sugar-like inositol, and the amino acid serine. However, a new study just published in *PLOS Biology* by Ruben D. Arroyo-Olarte, Nishith Gupta, and colleagues describes a new addition to this repertoire, and in a rather unexpected place—in the membranes of a widespread and occasionally lethal opportunistic pathogen of humans and other animals, *Toxoplasma gondii*.

*T*. *gondii*, affectionately known as “Toxo”, belongs to the protozoan phylum Apicomplexa, which comprises more than 5,000 parasitic species, including the well-known human parasites that cause malaria and cryptosporidiosis. It’s estimated that approximately one-third of the global human population has been exposed to Toxo. In the very young, and those with a compromised immune system, the parasite can wreak havoc, sometimes with fatal consequences.

The authors had set out to study how Toxo makes its membranes but encountered a surprise when assessing the most basic fact—the lipid composition of those membranes. Examination of the lipid composition showed many of the usual suspects, but also a new, unknown component that didn’t match any previously identified parasite lipids. Mass spectrometry revealed it to be an exclusive lipid, phosphatidylthreonine (PtdThr).

PtdThr closely resembles the near-universal lipid phosphatidylserine (PtdSer) except for a slightly bulkier amino acid (threonine instead of serine) as its head-group. PtdThr has been described only a handful of times in the literature, and then only as a trace component, presumed to arise by errors made by the enzyme that incorporates serine into PtdSer. In Toxo, however, PtdThr makes up a substantial proportion of the organism’s membranes—in fact, considerably more than PtdSer.

Mammalian cells usually produce PtdSer by using one of the other glycerophospholipids as a starting material, and then simply exchanging the choline or ethanolamine in the head-group for serine. The enzyme that catalyses this reaction, PtdSer synthase (PSS), is found across the eukaryotic tree of life, including Toxo and humans. However, the authors found that the genomes of Toxo and a few other protozoans encoded a second closely-related enzyme with chemically-significant amino acid differences in its catalytic site.

The obvious inference was that these variant enzymes were PtdThr synthases (PTSs). To confirm the notion, the authors disrupted the PTS gene in Toxo, finding that this wiped out all PtdThr synthesis; showing that PTS is entirely responsible for making PtdThr; reintroducing an intact PTS gene gratifyingly reinstated PtdThr synthesis.

But does PtdThr matter for Toxo? The authors compared how the PTS mutant and the rescued strain fared in cultured human fibroblast host cells. Normally, the Toxo infection results in clear patches of host cell destruction called plaques, which in essence represent successive lytic cycles. Mutating the PTS gene dramatically reduced the size and number of plaques, while reinstating the functional enzyme rescued this effect. To find out why Toxo needed the exotic lipid, the authors dissected the mutant’s poor growth phenotype. Unexpectedly, the authors discovered that the parasite replication was normal; however, the exit from host cells was impaired, and invasion of new host cells was also compromised (Fig 1). Consistently, calcium-regulated gliding movement of the parasite that is needed for both entry and exit was halved. Because PtdThr’s close cousin PtdSer has been described to regulate calcium storage and signaling, the authors speculate that Toxo may have evolved PtdThr as a specialized variant of PtdSer to modulate calcium during its entry and exit to and from host cells.

**Fig 1:**
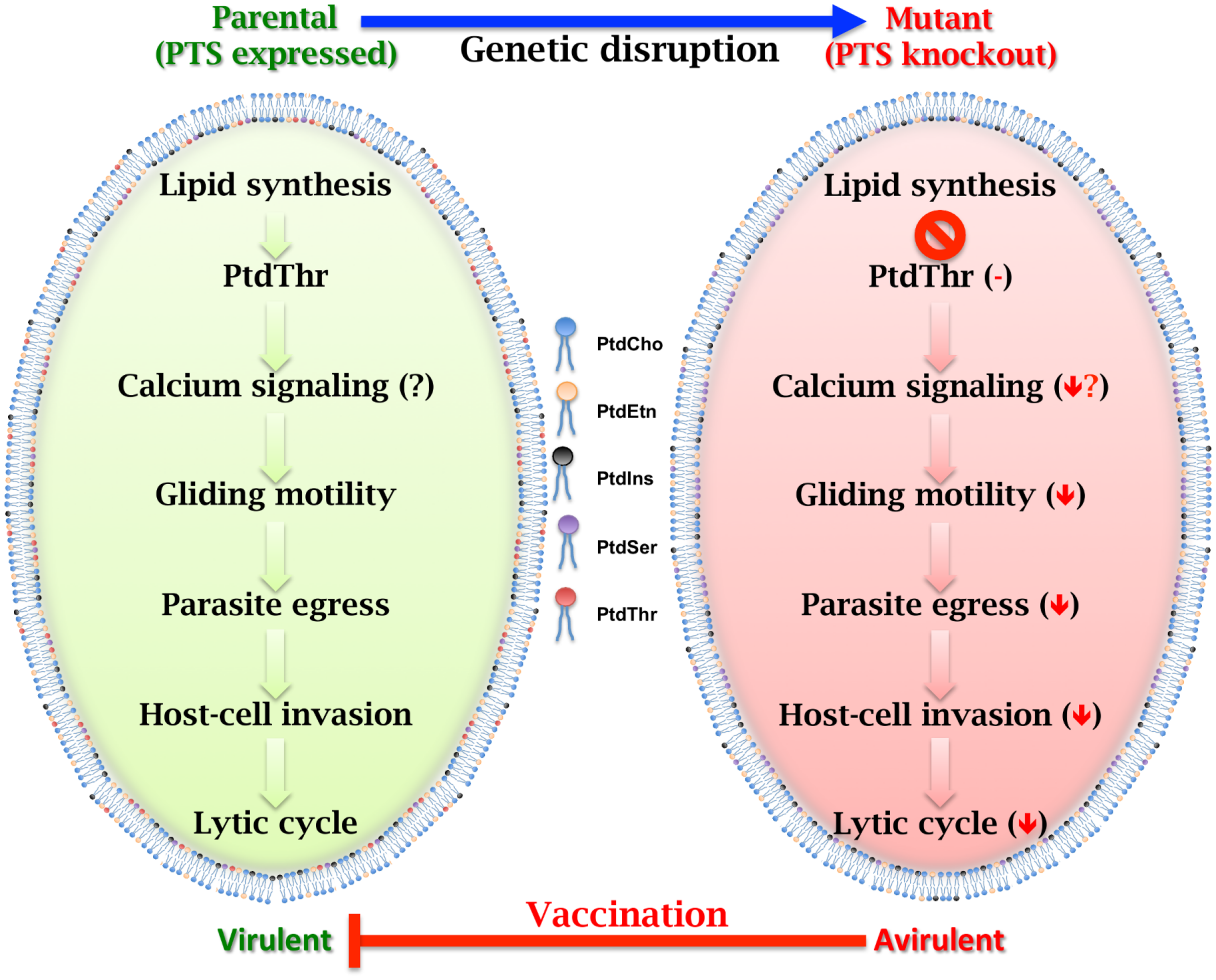
Phosphatidylthreonine-mediated control of lytic cycle and virulence in *T*. *gondii*. The parental strain of *T*. *gondii* expresses PTS, which produces PtdThr. Genetic disruption of PTS results in parasites that are unable to make PtdThr. Lack of PtdThr in the mutant compromises its gliding motility, which blights the sequential events of exit from parasitized cells, and entry into new host cells. Consequently, the PtdThr-deficient strain displays a severely impaired lytic cycle in human cells. Moreover, the mutant is highly attenuated in mice and confers potent immunity against hypervirulent and cyst-forming strains of *T*. *gondii*. *Image credit*: Nishith Gupta.

To see whether the defects observed in a culture dish are relevant to a real-life Toxo infection, the authors injected mice with the PTS mutant parasites. Not only were the mutant Toxo completely avirulent but they also acted as an effective metabolically-attenuated vaccine, conferring 100% resistance to subsequent challenge infection by parasite strains that normally cause acute and chronic infections.

In a nutshell, the authors show that an unusual lipid, previously only found in trace amounts in nature, is highly abundant in the membranes of a clinically important and widespread parasitic protist. Interestingly, for a lipid that is clearly crucial for the lifecycle and virulence of Toxo, the parasite has not only evolved a special enzyme to make PtdThr, but possibly also a pathway to make its own threonine—an amino acid that we humble humans have to obtain from our diet.

Toxo is likely not alone in its idiosyncratic lipid requirements. The presence of closely related PTS sequences in other globally prevalent apicomplexans like *Eimeria* and *Neospora* species and in more distant organisms, such as *Phytophthora infestans* (the critter responsible for the great Irish potato famine) suggests that PtdThr may not be as exotic as we previously believed. In Toxo and its parasitic relatives, the dependence of these common pathogens on an enzyme and lipid that their hosts lack opens up a potential opportunity for drug and vaccine development to target this Achilles heel.

## Abbreviations

PSS: PtdSer Synthase
PtdSer: phophatidylserine
PtdThr: phophatidylthreonine
PTS: PtdThr Synthase
